# Transmission characteristics of all-dielectric guided-mode resonance filter in the THz region

**DOI:** 10.1038/s41598-018-31931-3

**Published:** 2018-09-11

**Authors:** Hyeon Sang Bark, Geun Ju Kim, Tae-In Jeon

**Affiliations:** 1 Korea Maritime and Ocean University, Electrical and Electronics Engineering, Busan, 49112 South Korea; 2 Korea Electrotechnology Research Institute, Advanced Medical Device Research Division, Ansan, 15588 South Korea

## Abstract

In this study we report the first on the terahertz (THz) transmission characteristics of a guided-mode resonance (GMR) filter made of all-dielectric material. Two strong transverse electric (TE) resonance modes, TE_0,1_ and TE_1,1_, and one strong transverse magnetic (TM) resonance mode, TM_0,1_, were detected. The measured resonances can be explained by diffraction from the grating surface of the GMR filter, and by guiding along the inside of the filter (slab waveguide). Because two identical GMR filters were employed to overcome limited grating numbers, the measured Q-factors of the TM_0,1_, TE_1,1_, and TM_0,1_ modes were as high as 62.9, 71.0, and 74.4 respectively. Also, we obtained polarization efficiencies of up to 96.9, 96.3, and 92.9% for the TM_0,1_, TM_1,1_, and TM_0,1_ modes, respectively, when the GMR filter was rotated to 90°. By increasing the incident THz beam angle, one TE resonance can be divided into two TE resonances, and the resonant frequency can be adjusted like a THz tunable resonance filter. Furthermore, when the GMR filters were inserted between Teflon plates, only the TM_1,1_ mode was perfectly removed. The designed GMR filter has a high Q-factor, tunable filter, good polarizer, and good modulator characteristics. These experimental results were in good agreement with simulation results.

## Introduction

The thin metallic or dielectric patterns on a frequency-selective surface (FSS) can reflect and transmit specific electromagnetic fields. For this reason, the FSS has been commonly used in applications at microwave frequencies, for example, as the screen on a microwave oven^1^, in antenna radomes^2^, and communication filters^3^, and also in the optical region, as filters^4,5^ and polarizers^6^. A typical FSS is comprised of metamaterials which have a thin and repetitive metallic pattern on their dielectric surface, and have been widely studied recently. Metamaterials for THz frequencies have been suggested as ideal choices for good performance filters^7^, sensors^8,9^ and absorbers^10^ because of their high Q-factor^11–13^. Recently, metallic THz metamaterials, designed with slits to enhance the THz field, have been introduced for specific frequency band transmission at high THz^14,15^. These THz metamaterials have only been fabricated in metal. Creating metal patterns is a complicated process with high cost. At the same time, the high refractive index of the metal also results in high reflective loss. This high reflection loss limits the applications of metamaterials.

Meanwhile, polarizers^16^ and modulators^17^ for THz frequencies have been studied and applied in many fields, including imaging^18^, sensing^19^, and communication systems^20^. Carbon nanotubes and nanowires^21,22^, parallel-plate waveguides^23,24^ and metamaterials^25^ have been used to make polarizers and modulators which operate in the THz region. But because these devices are also complicated to fabricate and suffer high refraction loss, they have had limited applications. As an alternative to overcome these problems, we propose using a guided-mode resonance (GMR) filter. The main function of a GMR filter is to select a narrow frequency region with a very high Q-factor.

In this paper, an all-dielectric GMR filter operating in the THz region is proposed for the first time. The GMR filter is a combination of a grating on the filter surface and slab waveguides of the filter substrate. Previous studies have investigated the propagation of a THz wave through various structures, for example, metal gratings^26^, grooves^27^, and slab waveguides^28^. However, the characteristics of an all-dielectric GMR filter in the THz region have not been reported. Unlike metamaterials, an all-dielectric material such as quartz is efficient for measuring THz transmissions, because it has a lower refractive index and absorption^29^. For these reasons, strong TM and TE resonance modes can be detected. The designed GMR filter has an incident THz beam angle dependent tunable THz filter, a rotating GMR filter angle dependent THz polarizer, and uses the refractive indices of the incident material dependent THz modulator. The measurement results agreed very well with results from the simulations.

## Results

### GMR Phenomenon

THz time-domain spectroscopy (THz-TDS) was used to characterize the performance of the GMR filter. In our photoconductive THz-TDS system^23,27,30^, two GMR filters made of quartz (prepared by Buysemi Co. using an etching method) were located between two parabolic mirrors. Quartz has many advantages in THz GMR filter designs. First, due to its low refractive index and low absorption coefficient in the THz region^29^, it can reduce refraction and attenuation losses by the material. Second, samples can be produced with thicknesses of hundreds of microns, and with a grating pattern of tens of microns, because of the high strength of the material. Each grating pattern consists of a groove height (D1) of 60 *μ*m, substrate thickness (D2) of 168 *μ*m, period (∧) of 460 *μ*m, and a filling factor (F) of 32%. The separation between each of the grooves is 313 *μ*m. The THz wave is incident from the air onto the GMR filter (+y-direction).

**Figure 1:**
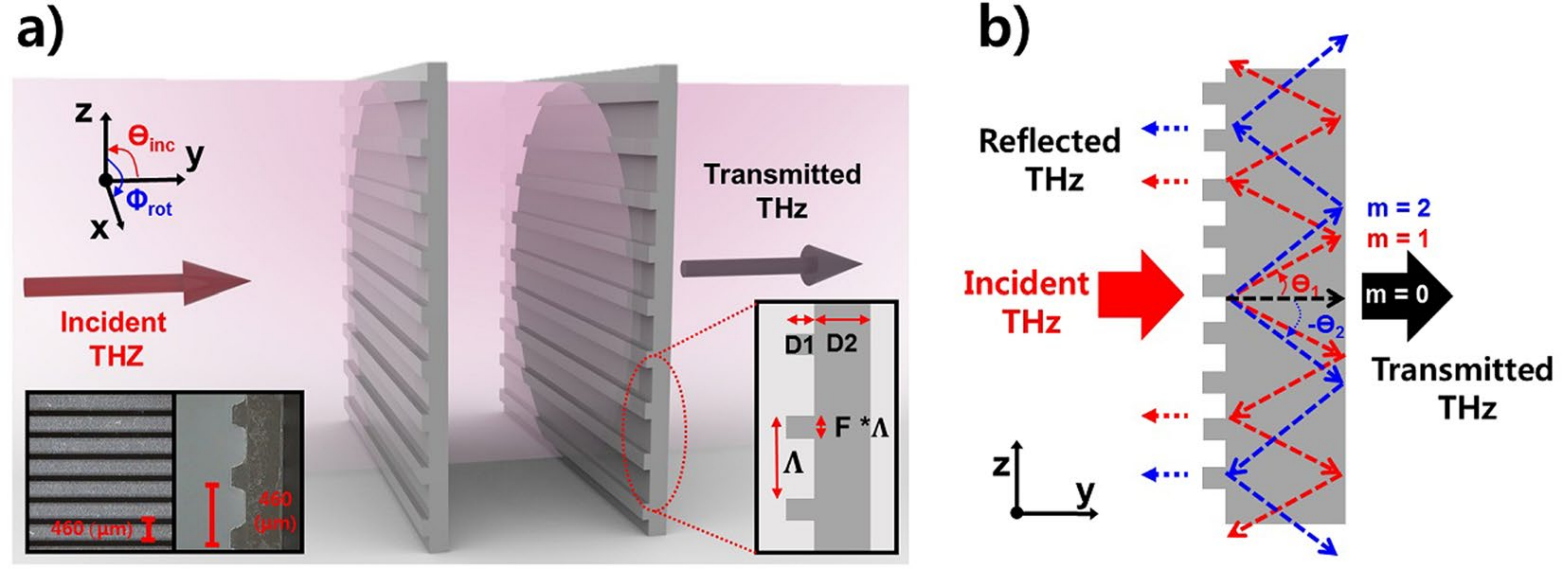
(**a**) Schematic diagram describing a THz beam passing through two identical GMR filters. The inserted photos show top-view and cross-sectional images of the GMR filter (quartz). Each GMR filter has a width of 30 mm and length of 30 mm. The two GMR filters are 15 cm apart from each other. The linearly polarized THz beam enters the GMR filter to a diameter of 25-mm. The separation between each of the grooves is 313 *μ*m (D1 = 60 *μ*m, D2 = 168 *μ*m, ∧ = 460 *μ*m, and filling factor = 32%). Each GMR filter has a total of 65 grooves. (**b**) Schematic diagram of a THz beam diffracted inside of the GMR filter.

Figure 1a shows a schematic of the experimental configuration for THz transmission measurements through two identical GMR filters. The two filters are exactly the same because each is made with the same process using a single mask. The two filters are spaced 15 cm apart and can easily be combined with other components. Since the THz beam passes through the two filters independently, the spacing and order of the two filters does not affect the results. The operation of a GMR filter can be explained by two physical mechanisms, diffraction and guiding. When a THz wave is incident on the GMR filter, diffraction is produced by the grating. If the angle of the diffracted mode produced by the grating matches the angle of a guided mode produced by the slab waveguide, the coupled THz wave propagates along into the slab. During the guiding process the coupled THz wave is gradually recombined in the grating and reflected toward the incident direction (−y-direction) as shown in Fig. 1b. On the other hand, the opposite surface of the grating has total internal reflection during the guiding process because of the difference in refractive index between the GMR filter and incident material (the surrounding media of the GMR filter). Therefore, there is no transmission at the opposite surface with respect to the frequency of the guided THz wave. This phenomenon creates a strong resonance in the spectrum. The incident angle of the electric field, *θ*_*inc*_, and the angle of the *m* th diffracted mode, *θ*_*m*_, can be determined by the well-known grating equation^31^.

1 $$\sqrt{{\varepsilon }_{avg}}\,sin{\theta }_{m}=\sqrt{{\varepsilon }_{inc}}\,sin{\theta }_{inc}-m\frac{c}{f\times \wedge },$$

where *ε*_*avg*_ is the average dielectric constant of the GMR filter, *ε*_*inc*_ is the dielectric constant of the incident material, *c* is the speed of light, *m* is the *m* th diffraction mode, and f is the frequency. In order to guide the diffracted electric fields within a slab, the effective dielectric constant of the guided mode has to be greater than the dielectric constant of the incident media and less than the average dielectric constant. Therefore, the conditions for GMRs are given by equation (2)^30–32^.

2 $$\sqrt{{\varepsilon }_{inc}}\le |\sqrt{{\varepsilon }_{inc}}sin{\theta }_{inc}-m\frac{c}{f\times \wedge }| < \sqrt{{\varepsilon }_{avg}},$$

**Figure 2:**
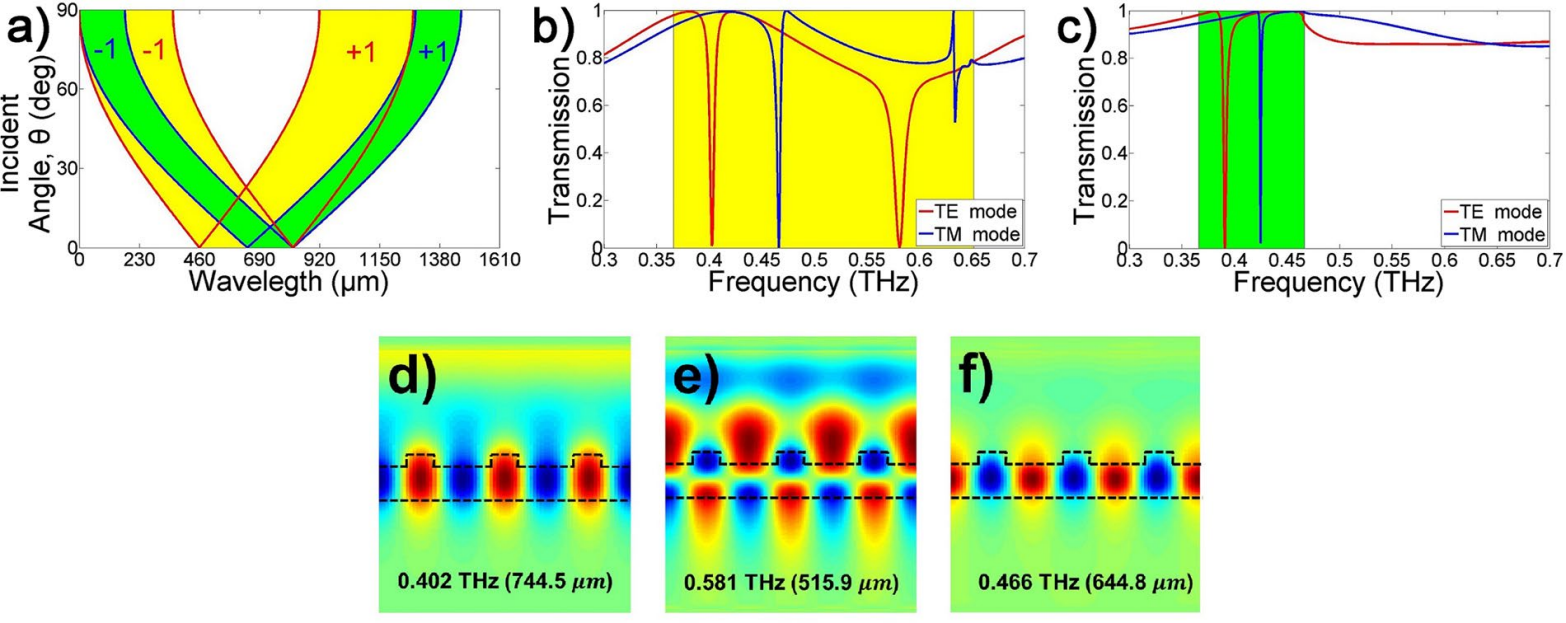
Resonance properties of GMR filter. (**a**) Regions of resonance in the GMR filter. The yellow and green areas indicate incident materials of air and Teflon, respectively. The transmission of TE (red line) and TM (blue line) resonance modes using an FDFD simulation when the incident angle is zero. (**b**) The incident material is air; and (**c**) the incident material is Teflon. The field distributions of resonances when the incident material is air: (**d**) Electric field of the TE_0,1_ mode; (**e**) electric field of the TE_1,1_ mode; and (**f**) magnetic field of the TM_0,1_ mode. Black dashed lines are outlines of the structure, with the three unit cells illustrated.

If the incident angle and period are fixed, the frequency (wavelength) range satisfying equation (2) exists between $\sqrt{{\varepsilon }_{inc}}\pm \sqrt{{\varepsilon }_{inc}}sin{\theta }_{inc}$ and $\sqrt{{\varepsilon }_{avg}}\pm \sqrt{{\varepsilon }_{inc}}sin{\theta }_{inc}$. The frequency (wavelength) range also depends on the dielectric constant of the incident material (*ε*_*inc*_). In this case, the satisfying range, ± of the diffraction mode (*m*), is symmetrically distributed, as shown in Fig. 2a. As the incident angle increases, one resonance is divided into two resonances to satisfy equation (2), and the larger the angle, the greater the spacing between the resonances. The yellow and green areas indicate the resonance regions at *m* = ±1 when the THz waves are incident on the GMR filter from the air and from Teflon, respectively. When a THz wave from the air is incident perpendicularly (*θ*_*inc*_ = 0) to a GMR filter made of quartz, which has a refractive index of 1.95 in the THz region, the first resonance (*m* = ±1) frequency is located between 0.3661 and 0.6517 THz. If the THz wave from Teflon, whose refractive index is 1.4 in the THz region, is incident perpendicularly to the same GMR filter, the range of the first resonance frequency is reduced from 0.3661 to 0.4665 THz.

Figure 2b shows the transmission of TE (red line) and TM (blue line) resonance modes using an Finite-Difference Frequency-Domain (FDFD) simulation for the designed single GMR filter with an infinite grating period and THz beam diameter, when the THz wave from the air is incident perpendicularly to the GMR filter. Two strong TE resonance modes appear at 0.402 and 0.581 THz, and one strong TM resonance mode appears at 0.466 THz. Because these resonance frequencies are sufficiently far away from the Rayleigh anomaly frequency ($c/(\sqrt{{\varepsilon }_{inc}})\times \wedge$) = 0.6517 THz)^33,34^, only the GMR effect can achieve the resonances with a high Q-factor. The yellow area indicates the only frequency range over which the first diffraction mode (*m* = ±1) can exist. Figure 2c shows the transmission when the incident material is not air but Teflon. Only one strong TE and TM resonance mode exists in the green region, which indicates that only the first diffraction mode (*m* = ±1) can exist.

When the incident material is air, the distributions of the electric field at the resonance frequencies of the TE mode are as shown in Fig. 2d,e. The electric field distributions of 0.402 and 0.581 THz across the filter are half and full wave loops, respectively. These electric field distributions indicate TE_0_ and TE_1_ modes, with the first subscript denoting the field distributions for guided modes in the slab waveguide. The second subscript of the mode expression denotes the number of the diffracted mode by the grating. Therefore, the first and second resonances can be expressed as TE_0,1_ and TE_1,1_. Figure 2f illustrates the magnetic field of the TM_0,1_ mode, which is a half wave loop, and the first diffraction mode. Like the TE_0,1_ mode, the field is guided within the slab and exhibits a strong resonance. Meanwhile, the electric fields at the resonance frequencies are diffracted by the grating and guided by the substrate (D2). Therefore, strong resonances appear in the transmission measurement. The electric fields of the resonances that cannot pass through the substrate are diffracted back into the air (−y direction) from the substrate.

### GMR Characteristics

**Figure 3:**
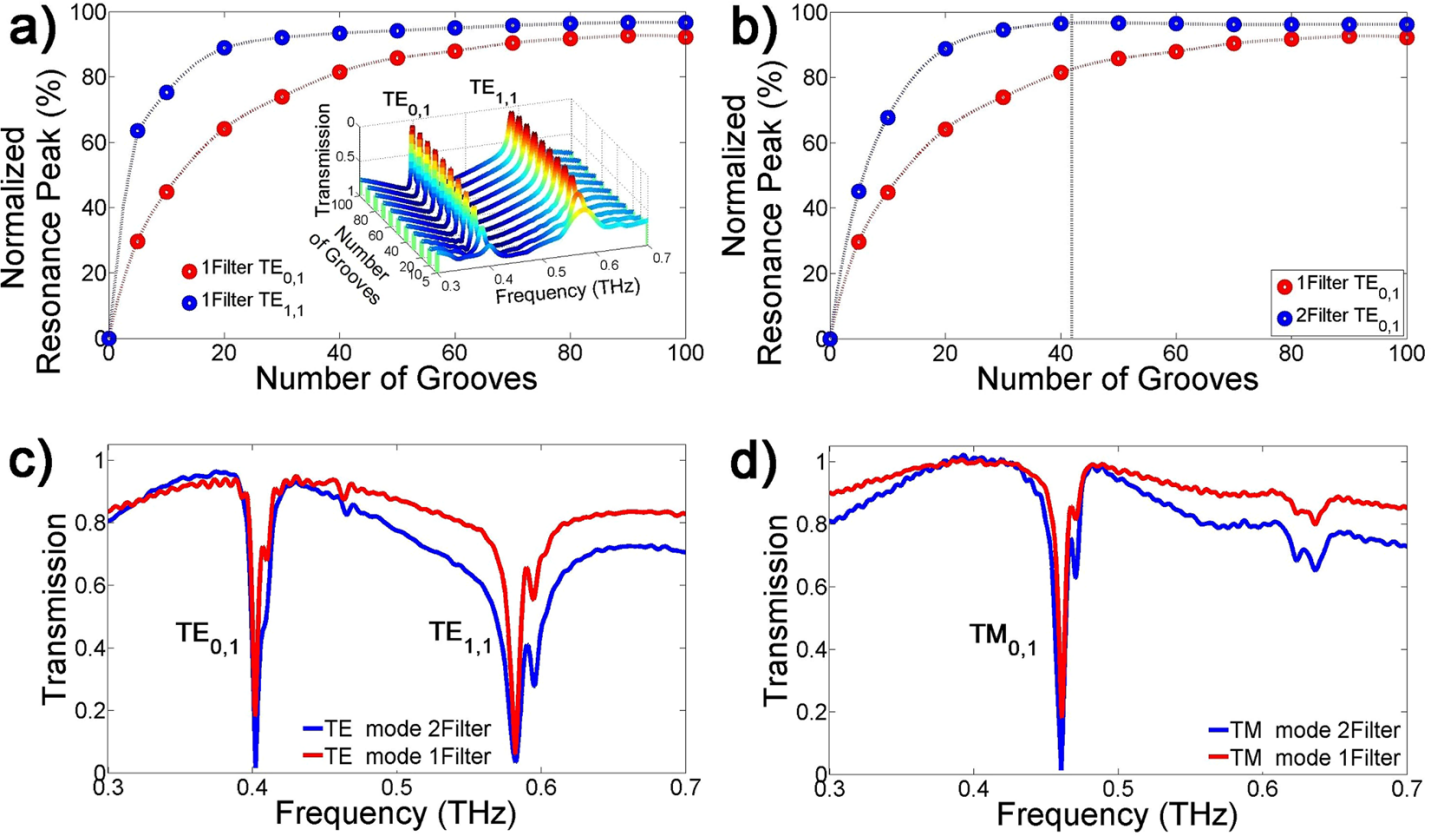
(**a**) Resonance peaks with varying number of grooves for the TE_0,1_ and TE_1,1_ modes. When the number of grooves is infinite, the resonance peak of transmission measurement is normalized to 100%. The inset figure shows simulated TE_0,1_ resonance modes with varying numbers of grooves. (**b**) Comparison of the TE_0,1_ mode for one and two GMR filters with varying numbers of grooves. The vertical dashed line indicates the effective number of grooves. (**c**) Comparison of TE resonance modes for one and two GMR filters. (**d**) Comparison of TM resonance modes for one and two GMR.

An ideal GMR filter requires an infinite number of grooves to achieve a strong resonance depth in the spectrum^35,36^. However, due to the limited THz beam diameter and GMR filter size, only a finite number of grooves are covered by the THz beam. Figure 3a shows the simulation results using FDTD for the resonance depth of the TE_0,1_ and TE_1,1_ modes with varying numbers of grooves. Because of the different transmission base lines, the resonance of the TE_1,1_ mode approaches 0 faster than that of the TE_0,1_ mode. The inserted figure in Fig. 3a shows the simulated transmission in a 3D graph, which shows that a large number of grooves exhibits higher and narrower resonance shapes than a small number of grooves. As the number of grooves increases, the resonance depths of both modes approach maximum^35,36^ (the transmission approaches to 0). In practice, because the number of grooves and the diameter of the THz beam cannot be infinite, we propose a method to improve the resonance depth by using two identical filters, as shown in Fig. 1a. Since the number of grooves in each filter is finite, the THz beam is not sufficiently filtered by the first filter. The unfiltered THz beam is filtered by the second filter so that the resonance depth is further enhanced. Using two filters has the effect of doubling the number of grooves. Figure 3b shows a comparison of the TE_0,1_ mode for one and two GMR filters with varying numbers of grooves. The normalized resonance peak of the two filters approaches the maximum value faster than just a single filter.

A comparison of the measured TE and TM transmission using one filter (red line) and two filters (blue line) is shown in Fig. 3c,d, respectively. We calculated the transmissions using the spectrum ratio of the output pulse with the GMR filters and a reference pulse without GMR filters. Because the THz beam is not perfectly perpendicular to the filter, a small side lobe resonance occurred at each main resonance. Due to the multiple reflections by the GMR filter, the base lines of the measured transmissions do not approach 1 at the measured spectrum, as shown in the simulation results in Fig. 2b. However, the resonance depths using the two filters almost approach maximum. The calculated Q-factors of the TE_0,1_, TE_1,1_, and TM_0,1_ modes were 62.9, 71.0, and 74.4, respectively, which are higher than that of normal metamaterials^13–15^. The transmission of the TE_0,1_ resonance mode using two filters was improved by about 14.1% compared with the transmission using one filter as shown in Fig. 3c. The resonance depths using the two filters approached 98.4%, which indicates that the effective number of the period of each filter is 42 out of a total of 55, as shown by the vertical dashed line in Fig. 3b. Because the THz beam is Gaussian distribution and circular profile, the 42 effective numbers are reasonable.

**Figure 4:**
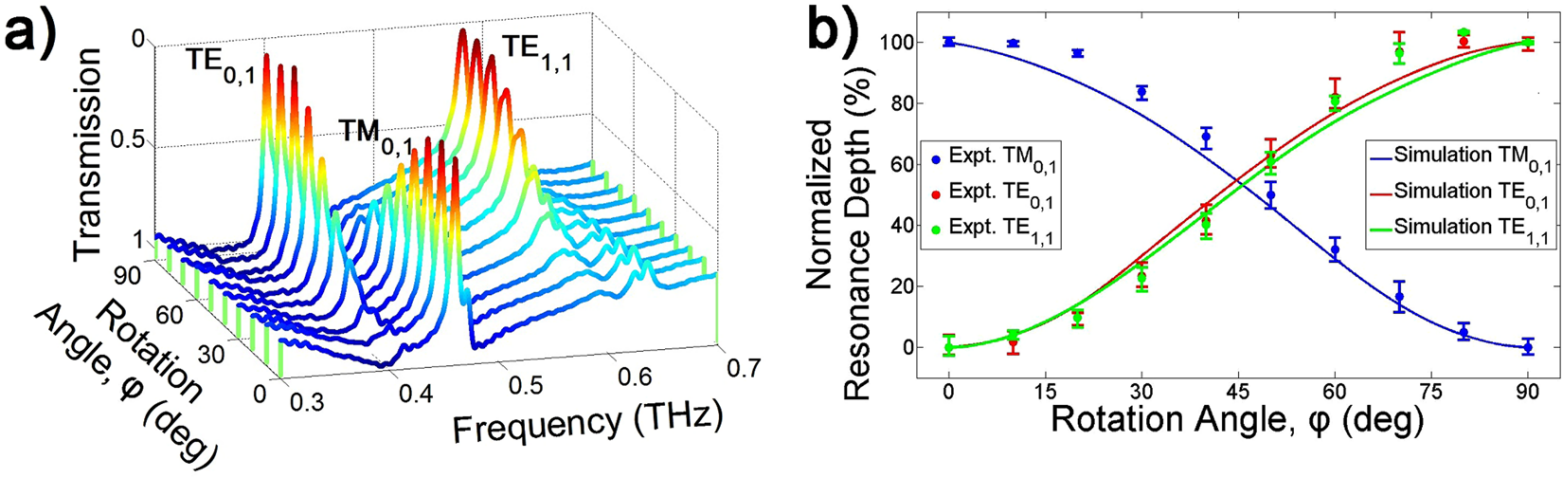
Polarization characteristic according to the rotation angle. (**a**) Measured transmission of the TE and TM modes with different rotation angles. (**b**) Normalized resonance depth for TE and TM modes. Dots and solid lines indicate the measurement and simulation fitting using Computer Simulation Technology (CST) software, respectively.

Since the proposed grating is asymmetrical in the xz plane, as shown in Fig. 1a, their resonance modes depend on the polarization of the incident beam. When the GMR filter is rotated from 0° to 90° for the THz wave polarization, as shown in the rotation angle (Φ_*rot*_) in Fig. 1a, the measured transmission spectra according to the rotation angle are as shown in Fig. 4a. To provide a 3D graph, the magnitude of transmission from 1 to 0 is shown. When the rotation angle is 0°, only the TM mode resonance exists because the grating direction and the THz wave polarization are perpendicular. However, by increasing the rotation angle, the resonance depth of the TM mode is reduced, and the resonance depths of the TE modes start to increase. The normalized resonance depth, which is the difference between the maximum and the minimum resonance depth at 0° and 90° converted to 100%, is shown in Fig. 4b with different rotation angles. The dots with error bars indicate those measurements which have a very small deviation. The solid lines indicate the simulation fitting using CST software. The measurement and simulation are in less agreement because the inner corners of the grating are not as perfect as in the simulations. However, when the angle increases, a superposition of the TM and TE polarized resonances can be obtained. When the angle is 45°, the resonance depths have about 50% TM and 50% TE modes. Finally, only the TE resonance modes exist at 90°. The polarization efficiencies for the measurement is in excess of 96.9, 96.3, and 92.9% for ${{\rm{TE}}}_{{\mathrm{0,1}}^{-}}$, ${{\rm{TE}}}_{{\mathrm{1,1}}^{-}}$, and ${{\rm{TE}}}_{{\mathrm{0,1}}^{-}}$ polarized THz waves, respectively. These high efficiencies demonstrate the very good characteristics of THz resonant polarization.

Meanwhile, the frequency range where the resonance exists is determined by the sine value of the incident angle, as shown in equation (2). If the THz beam is perpendicularly incident (*θ*_*inc*_ = 0) on the surface of the GMR filter, the positive and negative diffraction angles (*θ*_*m*_) of each high order mode are the same as shown in Fig. 1b. When the incident angle is increased, the positive and negative diffraction angles (${\theta }_{{m}^{+}}$ and ${\theta }_{{m}^{-}}$) of each mode are different for the normal direction of the filter surface. For example, the positive and negative diffraction angles for the first mode can be expressed as ${\theta }_{{1}^{+}}$ = *θ*_1_ + *θ*_*inc*_ and ${\theta }_{{1}^{-}}$ = *θ*_1_ − *θ*_*inc*_, respectively, where *θ*_1_ is the first diffraction mode angle at *θ*_*inc*_ = 0. Since these increased or decreased diffraction angles do not satisfy the guiding condition in the slab waveguide, high or low resonance frequencies are required to compensate for the incident angle. Therefore, the original resonance THz frequency is shifted to higher or lower frequencies to satisfy the guiding condition.

**Figure 5:**
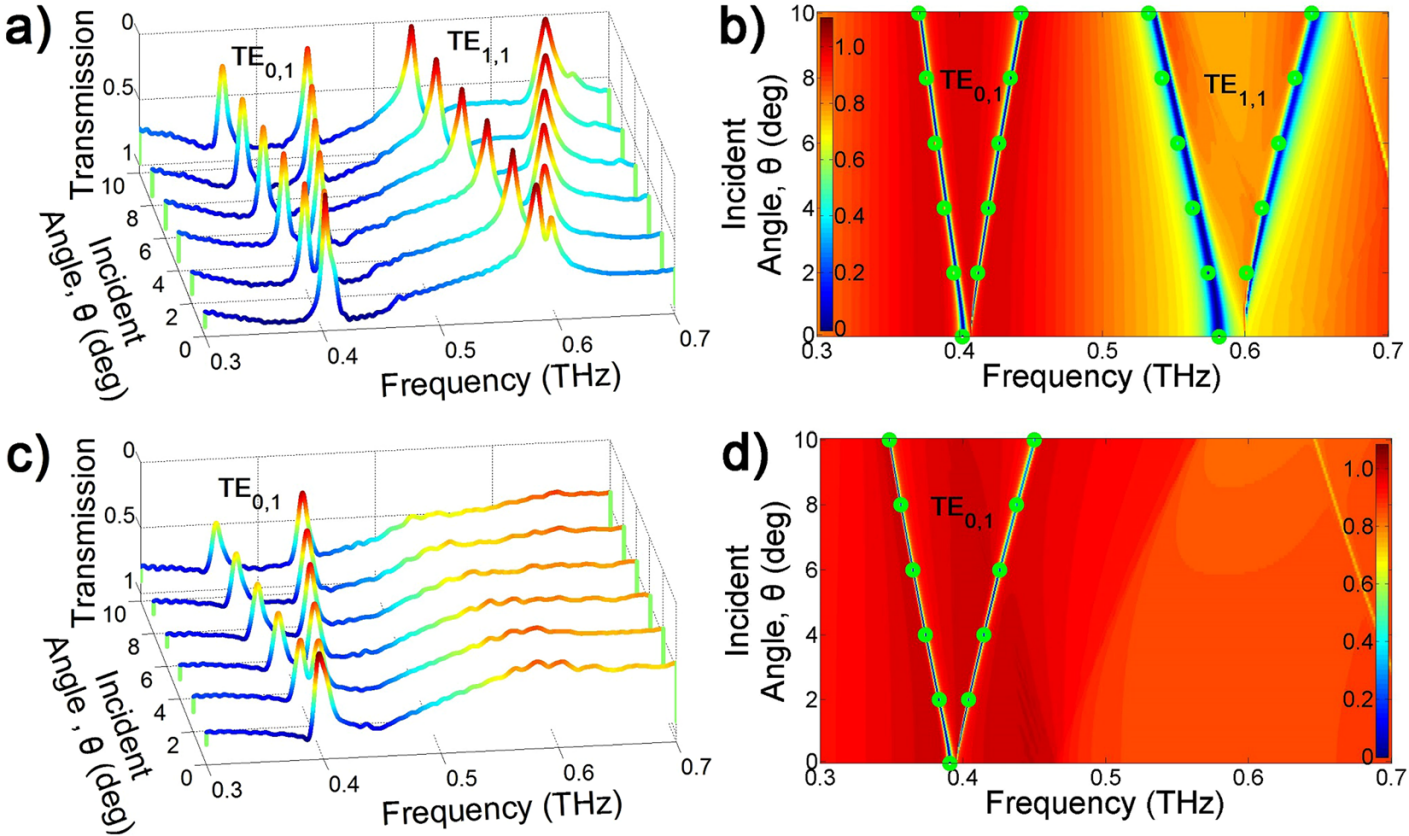
Tunable resonance filter of the TE mode according to the incident angle when the incident material is air. (**a**) Measurement; (**b**) 2D image of the FDFD simulation and dots indicate measured data within ±1.2 GHz variation. Modulation of the TE mode according to the incident angle when the incident material is Teflon: (**c**) Measurement; (**d**) 2D image of the FDFD simulation and dots indicate measured data within ±2.5 GHz variation. The vertical color bars indicate the intensity of the electric field.

As the incident angle increases from 0° to 10°, each TE resonance mode is divided into two resonances as shown in Fig. 5a. Like a THz tunable filter, these resonance frequencies depend on the incident angle. The 2D image of the FDFD simulation in Fig. 5b clearly shows the split resonances. The diffraction angle is proportional to the wavelength, and the sine value of the diffraction angle determines the range of the split resonances. Therefore, the resonances with a long wavelength (low frequency as TE_0,1_) have a smaller separation range than the resonances having a short wavelength (high frequency as TE_1,1_). The dots in Fig. 5b indicate the resonance frequencies measured with different incident angles. The measurements and simulations are in very good agreement.

When the THz wave is incident perpendicular to the GMR filter, the maximum and minimum frequencies at which resonance can exist depends on the refractive indices of the incident material (surrounding media) and the substrate, respectively. Because the THz wave is incident from air to quartz, the range is from 0.3661 to 0.6517 THz, as shown in the yellow area of Fig. 2a,b. The TE_0,1_, TE_1,1_, and TM_0,1_ modes with frequencies 0.402, 0.581, and 0.466 THz, respectively, are in this range. When two GMR filters are inserted between the three Teflon plates, the resonance range is reduced from 0.3661 to 0.4665 THz as shown in the green area of Fig. 2a,c. The minimum frequency of the range is the same because the refractive index of the substrate was not changed. However, when the refractive index of the incident material is changed from air to Teflon, the maximum frequency of the range is changed from 0.6517 to 0.4665 THz. Thus, only the TE_0,1_ and TM_0,1_ modes, whose resonance frequencies are 0.391 and 0.425 THz, can satisfy the new GMR conditions.

Figure 5c,d shows the measurement and FDFD simulation in which the thickness of the Teflon plates is 15 mm. The resonance depth and width of the TE_0,1_ mode are reduced and widened, respectively, as compared to the case without the Teflon plates, because of the absorption and dispersion by the Teflon. However, the TE_0,1_ mode is completely removed from the spectrum, as shown in the measurement and simulation. In addition, because the incident dielectric constant is changed from air to Teflon, the resonance dip frequency of the TE_0,1_ mode is also shifted from 0.402 to 0.391 THz. It can be used as a very good THz modulator and sensor.

## Discussion

The GMR filter is a useful device that can be used to overcome the disadvantages of metallic FSSs in the THz frequency region. For example, a metamaterial with a metal pattern on a dielectric substrate has a large reflection loss due to the metal pattern. This low transmission efficiency restricts its applications. However, the proposed GMR filter can reduce reflection and attenuation losses because it is made of an all-dielectric material, such as quartz. It can also be made from other dielectric materials without metal patterns on the surface, including Teflon, silica, Polyethylene, etc., which have a low refractive index and low absorption coefficient in the THz region.

The main parameters determining the resonance frequency are the refractive index, grating period, and thickness of the GMR filter, which are determined by the filter itself. These parameters determine the resonance frequency by changing the diffraction angle and length within the filter. However, other parameters such as the incident angle of the THz wave and the refractive index of the incident material also change the GMR conditions as shown in equation (2). We also demonstrated that the limited resonance depth resulting from the finite grating periods can be overcome by using two identical GMR filters. By performing the filtering process twice, it was possible to obtain almost the same effect as an infinite grating period filter. We were able to obtain a Q-factor of up to 74.4 for the TM_0,1_ mode. Because the Q-factor achieved by the GMR filter is higher than that for THz metamaterials, it can be used to replace applications currently using metamaterials. When the grating structure of the GMR filter was 45° (rotation angle) to polarize the THz beam, the resonance amplitudes of the TE and TM resonances became 50% and 50%, respectively. Also, we obtained polarization efficiencies of up to 96.9% for the TE_0,1_ mode when the filter was rotated to 90°. It was found to be a very good THz polarizer.

Meanwhile, wave guiding within the filter can only be achieved when the effective dielectric constant of the guided mode is greater than that of the incident material and less than that of the filter. Therefore, the positions of the TE resonance modes are separated when the incident angle is increased. When the incident angle was 10°, the separated frequencies of the resonance were 0.066 and 0.103 THz for the TE_0,1_ and TE_1,1_ modes, respectively. Furthermore, if the GMR filter is inserted between Teflon plates, the frequency range in which the resonance exists can be reduced. As a result, the TE_1,1_ mode can be perfectly removed. This serves as a good THz frequency modulator.

In conclusion, because the proposed GMR filter has a high Q-factor, tunable filter, good polarizer, and good modulator characteristics, it has potential for THz applications in spectroscopy, display, image sensor, and biomedical technologies in the future.

## Methods

### THz-TDS system

We fabricated a conventional THz time-domain spectroscopy system using a Ti:sapphire femtosecond laser (Mai Tai, Spectra Physics, USA), providing a 790 nm center wavelength, 60-fs duration at a 80-MHz repetition rate in a beam, with an average power of 12 mW on both the transmitter (Tx) and the receiver (Rx) antennas. The Tx antenna, consisting of coplanar 10-*μ*m wide metal lines with a separation of 80 *μ*m, was fabricated on a semi-insulating gallium arsenide (SI-GaAs) wafer. The laser excitation beam was focused onto the metal-semiconductor interface of the positively biased (80 V) transmission line. The Rx antenna, consisting of two 20-*μ*m wide stubs separated by a 5-*μ*m gap in a coplanar transmission line of two parallel 10-*μ*m wide metal lines with a separation of 30 *μ*m, was fabricated on a low-temperature-grown gallium arsenide (LI-GaAs) wafer. In our photoconductive THz-TDS system, two GMR filters made of quartz were located in between two parabolic mirrors for transmission measurement. We calculated the transmissions using the spectrum ratio of the output pulse with the GMR filters and a reference pulse without GMR filters.

### Numerical analysis

The numerical simulations are performed by the commercial software, CST Microwave Studio (2017 version) and by custom made FDFD simulation codes. The simulation in Fig. 4b was performed by the calculations of the CST transient solver, which is based on the finite-integral technique (FIT) method. A hexahedral mesh and periodic boundary conditions (PBC) were applied. Other simulations are performed by FDFD calculations. The transmission and reflection directions were set to total-field and scattered-field. The periodically repeating GMR structures were modeled by infinitely repeating GMR structures, by applying periodic boundaries in both directions.
